# Ppb-Level Self-Calibrating Ozone Detection Using a T-Type Multipass Enhanced Photoacoustic Sensor with a 9.46 μm Quantum Cascade Laser

**DOI:** 10.1021/acs.analchem.4c04999

**Published:** 2025-02-04

**Authors:** Yu-Xuan Wu, Pei-Ling Luo

**Affiliations:** † Institute of Atomic and Molecular Sciences, Academia Sinica, Taipei 106319, Taiwan; ‡ Department of Chemistry, National Taiwan Normal University, Taipei 11677, Taiwan

## Abstract

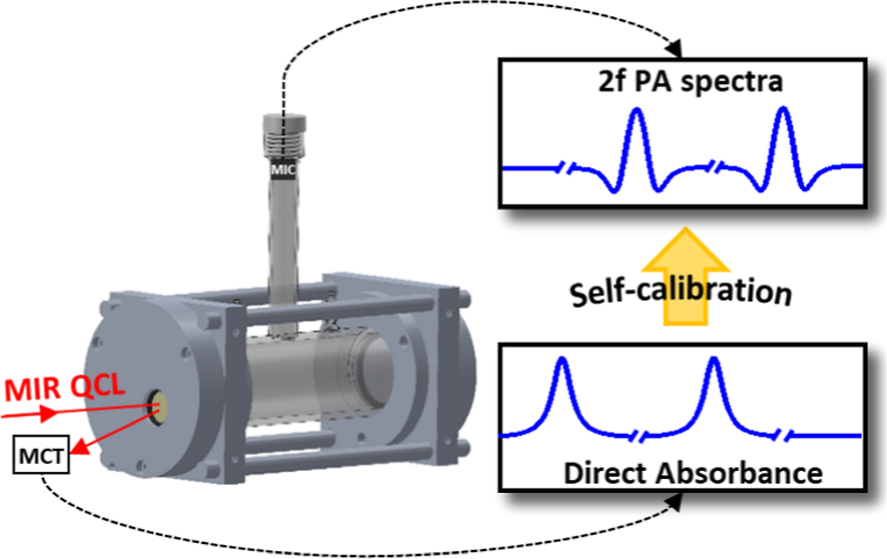

A sensitive, real-time, and accurate ozone (O_3_) sensor system is developed based on the combination of multipass enhanced photoacoustic (MPPA) and direct multipass absorption spectroscopy with a mid-infrared quantum cascade laser (QCL). The QCL with an emission wavelength of 9.46 μm was used to probe the O_3_ absorption lines without interference from the absorption of water and carbon dioxide in the flowing mixtures. The MPPA sensor was constructed with a T-type cell composed of a vertical cylinder and a horizontal cavity which were designed as an acoustic resonator and for multipass absorption enhancement, respectively. By periodically on–off switching the modulation of the laser wavelength, rapidly switched measurements of direct absorption and PA spectra can be achieved for real-time and accurate calibrations of the second harmonic (2f) PA signals with the direct absorbance spectra of O_3_. Moreover, a detection limit of O_3_ of 6 ppb at an average time of 300 s was achieved, and a short sensor response time of 16 s was also obtained in the flow mixtures with a flow rate of 50 sccm. This work provides a reliable method for O_3_ detection with capabilities of parts-per-billion-level sensitivity and on-site real-time concentration calibration, thus holding promise for in situ ozone monitoring under various environments.

As one of the most important greenhouse gases and short-lived climate pollutants, ozone (O_3_) plays an important role in the effects of global warming as well as in influencing the abundance of tropospheric OH radicals and further affecting the atmospheric oxidizing capacity and air quality.^1−4^ In the Earth’s troposphere, ozone is mainly generated through the photochemical reactions involving HO_2_, RO_2_, NO_*x*_, and volatile organic compounds (VOCs),^1,2^ and its abundance is sensitively influenced not only by different meteorological factors such as solar radiation, wind, and humidity but also by human activities. Moreover, ground-level ozone pollution recently has been considered as an important issue detrimental not only to human health but also threatens the populations of birds and staple crops.^5,6^ According to field observations, the ozone level may widely vary from a few ppb to over 100 ppb during 1 day in the outdoor^7−9^ and it can be observed up to a few hundred ppb in the indoor environments with air purifiers.^10^ Currently, the World Health Organization (WHO) guidelines suggest that the allowable ozone level is 60 ppb for an exposure period of 8 h.^7^ On the other hand, as a key oxidizing agent, ozone is also widely adopted with a concentration range from hundred ppb to ppm levels in the chamber experiments of gas-phase oxidation.^11,12^ Owing to the high reactivity of ozone, sensitive and dynamic ozone detection would be critical and essential in both field measurements and simulation chamber experiments to further explore its implications on the global and regional atmosphere.

Up to now, various analytical approaches including indirect methods with metal semiconductor or amperometric-based sensors as well as direct methods using ultraviolet (UV) or infrared (IR) absorption spectroscopy have been applied for ozone determination.^13−18^ With simpler constructions and lower costs, the indirect methods of ozone detection are widely used in field measurements;^13−15^ however, the indirect methods suffer from large uncertainties due to the strong interferences from humidity and other oxidizing species in ambient environments. In contrast, the optical detection of ozone with direct absorption spectroscopy can achieve accurate concentration measurements.^13,16−18^ Direct UV absorption spectroscopy-based ozone sensors are also widely used in many applications; nevertheless, they typically require an additional ozone scrubber to obtain the ozone concentration by the subtraction of the measured spectra with and without the scrubber, causing longer measurement time and additional uncertainties from the scrubber.^13,16^ In the recent few years, thanks to the emerging development of mid-infrared (MIR) lasers, trace gas sensors based on MIR absorption spectroscopy have become more powerful and are readily available to monitor and distinguish different trace molecules with their rotationally resolved absorption spectra.^19−24^ Additionally, several approaches such as coupling the MIR laser into a multipass absorption cell^19−22^ or employing wavelength modulation spectroscopy (WMS)^21^ and photoacoustic spectroscopy (PAS)^22−24^ have been reported to achieve gas sensing with sufficient sensitivity. Being a background-free technique, PAS has been reported to achieve ppb-level sensitivity with a rather short absorption path length compared to the direct absorption with WMS. For instance, recently, the real-time online detection of CO impurity concentrations in H_2_ has been demonstrated using a photoacoustic heterodyne sensor with a MIR laser at 4.61 μm^23^ and simultaneous determination of multispecies of CH_4_, N_2_O, and H_2_O has been achieved by means of MIR quartz-enhanced photoacoustic spectroscopy near 7.93 μm.^24^ In addition, the sensitivity of PAS can be increased by combination with the multipass enhanced approach. For instance, a Herriott-type multipass photoacoustic cell coupling with a high-power CO_2_ MIR laser has been proposed for in situ monitoring of trace gases such as ethane, methanol, and ethanol with sub-ppb level sensitivity.^22^ Because of the larger divergence angles of the CO_2_ MIR laser, the cell was designed with a large cell diameter to allow 36 multipasses of the MIR beams, causing a rather large cell volume of 2.3 L and a long response time of 5 min in the flow mode. Although the multipass enhanced photoacoustic (MPPA)-based sensors can be designed with more compact and small cell sizes, these systems have been achieved mainly in the near-infrared (NIR) region.^25−27^ In comparison to gas sensing in the MIR region, the molecular absorption cross sections in the NIR region are typically 3–5 orders of magnitude weaker than those in the MIR range; therefore, it would be difficult to use direct multipass absorption spectroscopy in NIR for self-calibrating the PA signals from the same module. As one of the laser-induced spectroscopic techniques, PAS generally requires additional concentration calibration using standard gas samples with known mixing ratios of the trace gas. Therefore, it still remains a challenge to use PAS to determine reactive molecules such as ozone, which are difficult to make the standard gas samples and cannot be stored for a long time.

In this work, a self-calibrating photoacoustic sensor system utilizing a 9.46 μm quantum cascade laser (QCL) combined with multipass enhanced photoacoustic (MPPA) spectroscopy is proposed to measure the ozone concentrations in ozone-air flowing mixtures. The photoacoustic sensor constructed with a T-type glass cell, which is composed of a vertical resonant cylinder for acoustic detection and a larger horizontal cavity for multipass absorption enhancement, enables sensitive ozone detections via direct absorption and photoacoustic signals with the same single sensor. The photoacoustic signals thus can be real-time and calibrated on-site with the direct absorbance spectra under various conditions. To our knowledge, this is the first time that an MPPA-based sensor has been demonstrated in the mid-infrared spectral range with both direct absorption and photoacoustic detection capabilities, indicating a great potential for photoacoustic gas sensing without an additional calibration system.

## Experimental Section

### Selection of the Optical Probing Range for Ozone Detection

**Figure 1:**
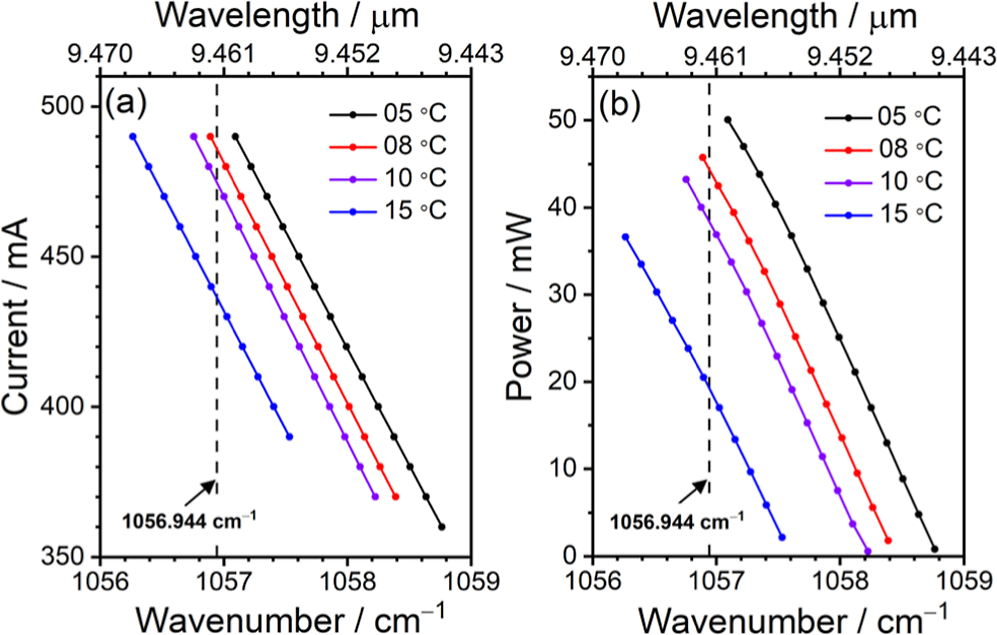
Dependences of the (a) employed current and (b) output power of the DFB–QCL on the wavelength. The temperatures of the laser module were set at 5 °C (black), 8 °C (red), 10 °C (purple), and 15 °C (blue).

**Figure 2:**
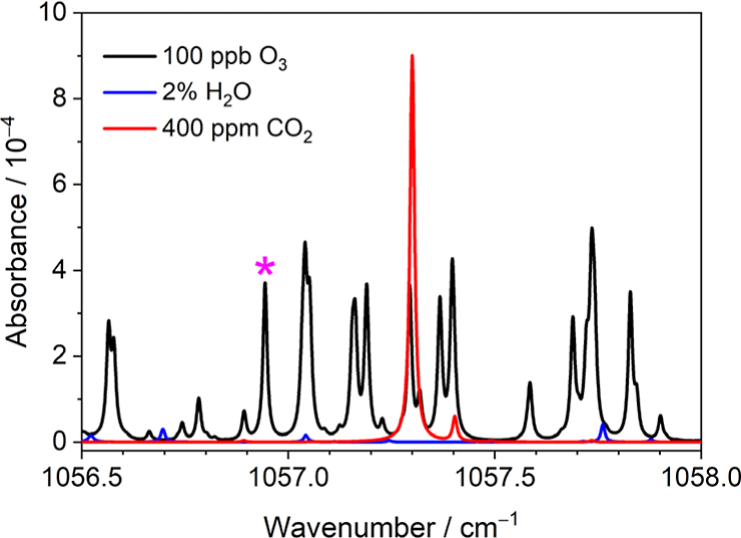
Absorbance spectra of O_3_ (100 ppb), H_2_O (2%), and CO_2_ (400 ppm) in the ambient air for an absorption path of 10 m, a pressure of 60 Torr, and 296 K. The marked star indicates the selected line at 1056.944 cm^–1^ for ozone detection.

Ozone (O_3_), as an asymmetric top molecule, has three fundamental vibrations, including symmetric stretching (ν_1_), bending (ν_2_), and asymmetric stretching (ν_3_) modes at 1103, 701, and 1042 cm^–1^, respectively.^28^ The ν_3_ vibrational mode of O_3_ has the strongest band intensity, and its rovibrational transitions are well characterized and listed in the HITRAN database.^29,30^ In this work, a continuous wave distributed feedback quantum cascade laser (DFB–QCL) was used as the probing beam for ozone detection near 9.46 μm (1057 cm^–1^). The wavelength of the QCL can be tuned by adjusting the current and temperature of the laser module, and its output power also depends on the used current and temperature, as shown in Figure 1. To implement O_3_ detection in ambient air, selection of the optical probing range is important to avoid interference from absorption of atmospheric CO_2_ and H_2_O. Figure 2 shows the absorbance spectra of O_3_ (100 ppb), H_2_O (2%), and CO_2_ (400 ppm) in the ambient air that were simulated for an absorption path of 10 m and at a pressure of 60 Torr and 296 K. By considering the available power of the QCL and the line strength of the O_3_ absorption lines, an O_3_ line, assigned to be the ν_3_ 23_0,23_–22_0,22_ transition, at 1056.944 cm^–1^ with a line strength of 3.45 × 10^–20^ cm molecule^–1^ was hence selected for O_3_ detection.

### T-Type Multipass Enhanced Photoacoustic (MPPA) Sensor and Ozone Generator

**Figure 3:**
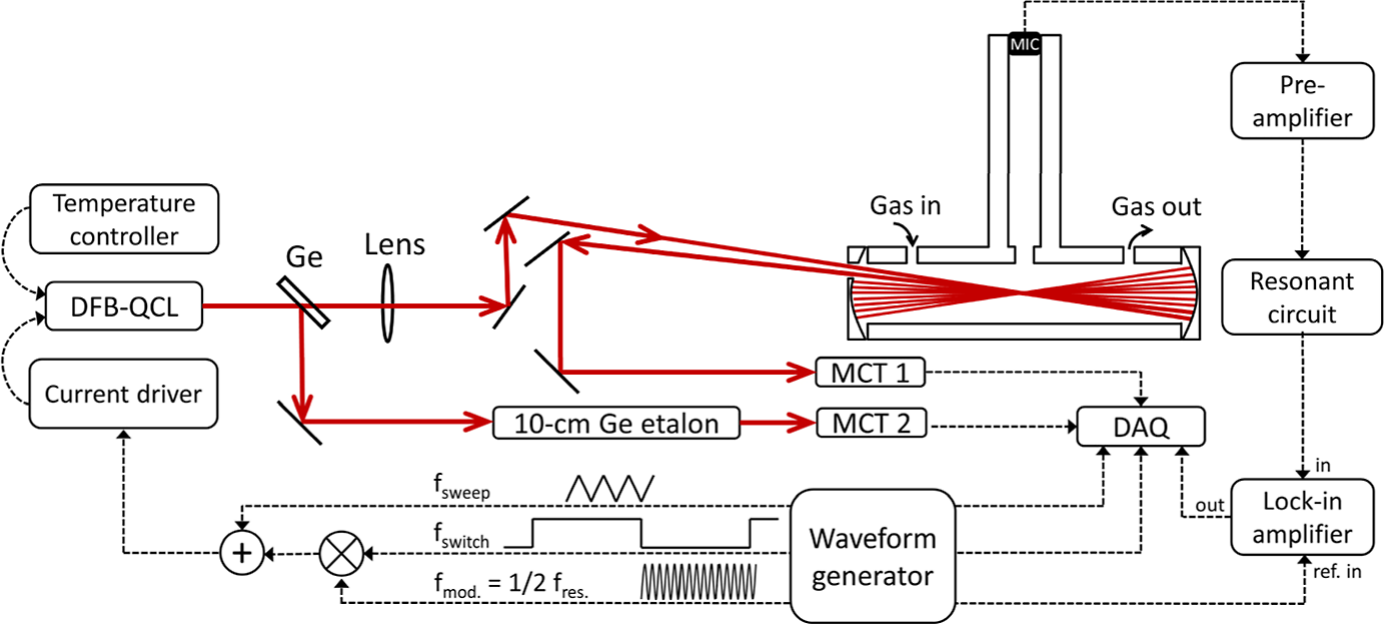
Schematic of the experimental setup for O_3_ detection with the T-type multipass enhanced photoacoustic sensor. The acoustic receiver includes the MIC circuit, the preamplifier, and the resonant circuit, as shown in Figure S1. Here, DFB–QCL is the distributed feedback quantum cascade laser, Ge is the germanium window as the beam splitter, MCT is the HgCdTe detector, DAQ is the data acquisition board, and MIC is the microphone.

**Figure 4:**
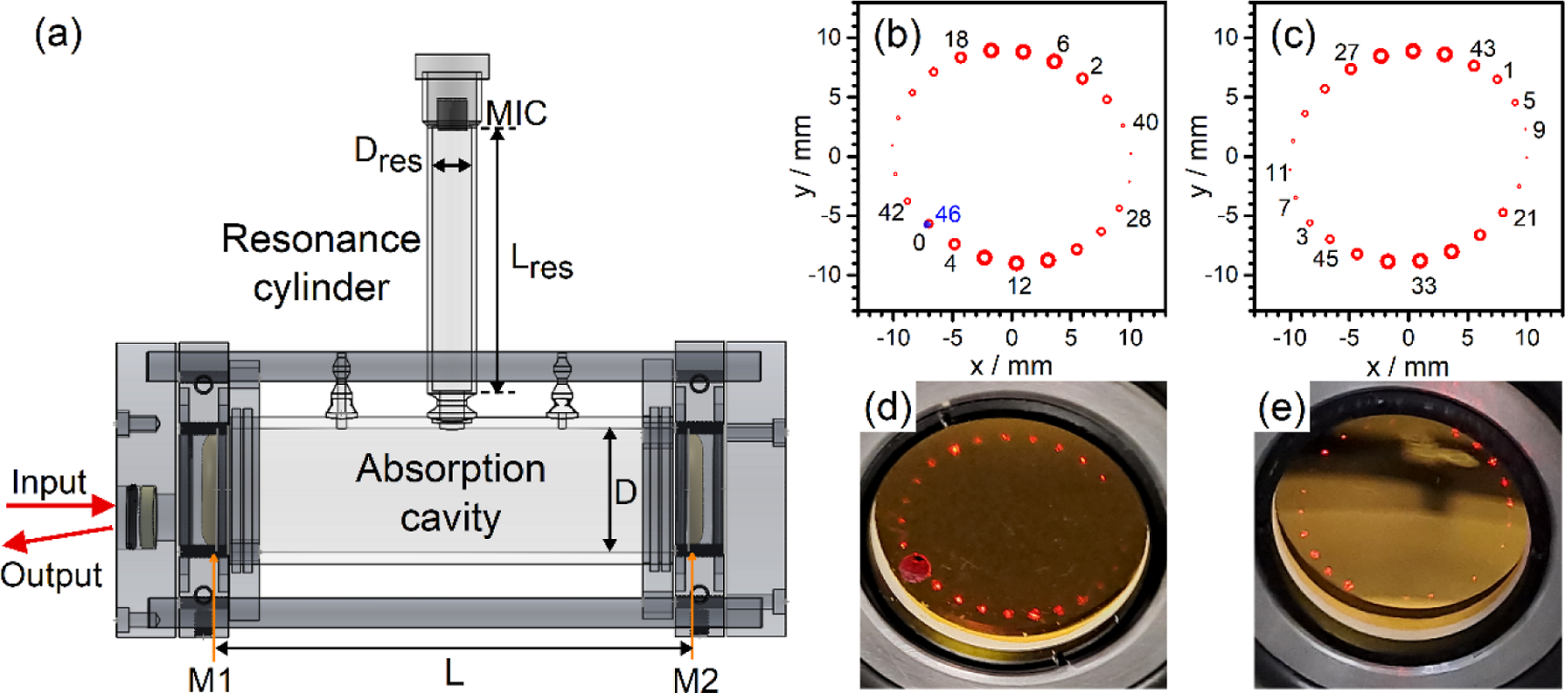
(a) Schematic diagram of the T-type multipass enhanced photoacoustic (MPPA) sensor. The simulated beam spot distributions on (b) the first concave mirror (M1) and (c) the second concave mirror (M2). Here, the numbers indicate the number of passes. The input (number 0) and output (number 46) beams were designed with the same position on the M1. Photographs of the beam spot pattern on the (d) M1 and (e) M2 with the red light alignment laser.

Figure 3 presents a schematic diagram of the experimental setup for O_3_ detection of _3_ with the multipass enhanced photoacoustic (MPPA) sensor. The DFB–QCL (Alpes Lasers) was set near 9.46 μm (1057 cm^–1^) for probing the O_3_ absorption lines. The temperature of the laser was set at 8 °C. By sweeping the current from 480 to 493 mA, the laser frequency could be scanned from 1056.98 to 1056.87 cm^–1^ with an optical power of 44 mW. The laser output was split into two parts. One of the beams with an optical power of approximately 30.6 mW was sent to a T-type PA cell, and another beam was passed through a 10 cm germanium (Ge) etalon with a free spectral range of 0.0163 cm^–1^ for calibrations of the sweeping frequency range. The T-type PA cell consisted of a T-shaped glass tube and two concave gold mirrors (diameter: 25.4 mm; radius of curvature: 100 mm) with one off-axis hole (3 mm) on the first mirror (M1) for the input and output ports of the QCL beam, as shown in Figure 4. The glass tube as the main body of the sensor was chosen to reduce the ozone decomposition through heterogeneous reactions on the tube wall during the measurements.^31^ The T-shaped glass tube was constructed with a vertical resonant cylinder [inner diameter (*D*_res_): 8 mm; length of the resonant cylinder (*L*_res_): 60 mm] for photoacoustic detection and a horizontal cavity with an inner diameter (D) of 27 mm to allow the passage of the probe light with multiple passes. The volume of this T-type PA cell was estimated to be 56.8 mL.

**Figure 5:**
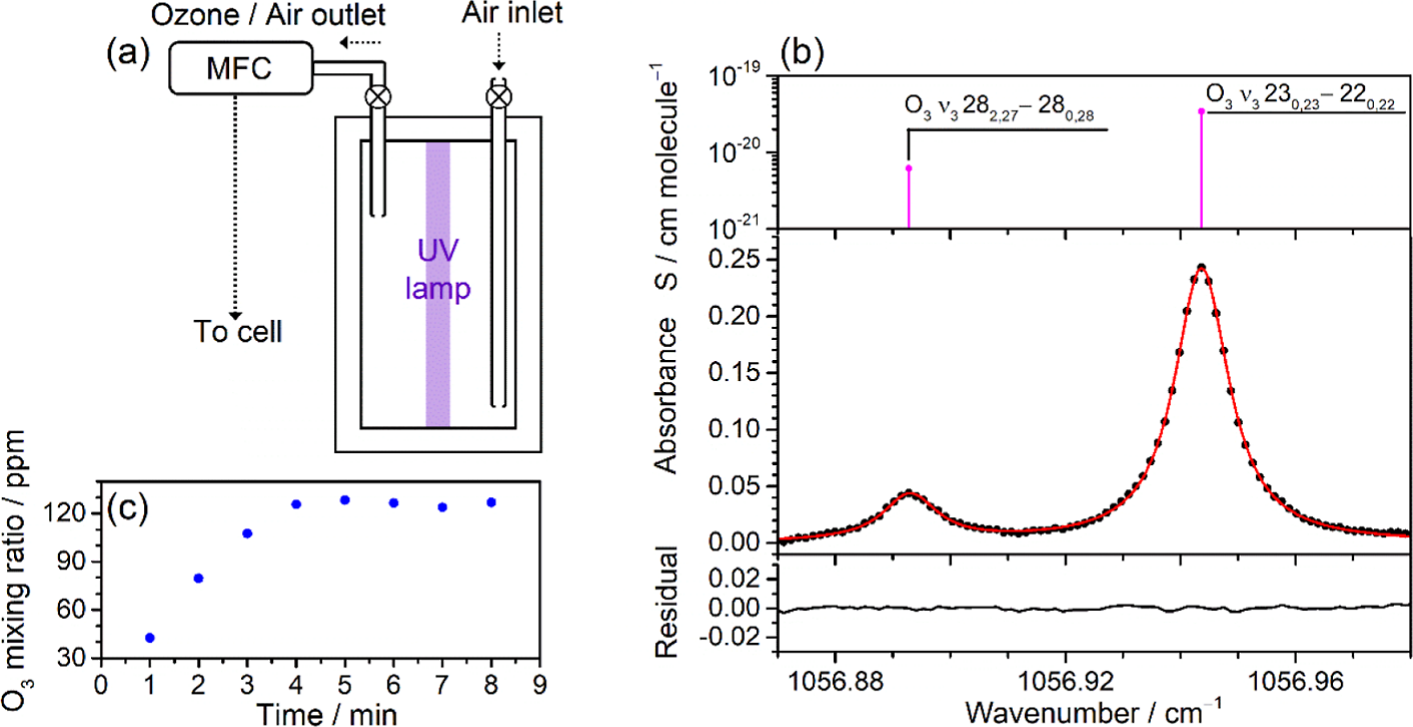
(a) Schematic diagram of the ozone generator. (b) Absorbance spectra of the O_3_ ν_3_ 23_0,23_–22_0,22_ and 28_2,27_–28_0,28_ transitions in the region of 1056.87–1056.98 cm^–1^. The black circle represents the measured absorbance signals, the red line represents the fitting curve, and the black line represents the fitting residual. The concentrations of ozone were determined to be 126 ppm by using the measured integrated absorbance area, absorption path, and the line strengths taken from the HITRAN database.^30^ (c) Mixing ratios of the generated ozone as a function of the used UV photolysis time in different preparation processes.

In addition, the sensor system was specially designed with a detachable T-shaped glass tube, allowing the user to easily work on a multipass alignment procedure of the laser beams and to clear the mirrors more conveniently when the central glass tube was uninstalled. Figure 4b,c displays the simulated beam spot distributions on the first concave mirror (M1) and the second concave mirror (M2), respectively. In the case without installation of the central glass tube, a red light laser was first used to achieve multiple reflections between two mirrors, separated by 94 mm (L), to produce 46 passes, resulting in a path with a total length of 438.4 cm. The input (number 0) and output (number 46) beams were designed with the same position on the M1. The off-axis hole on M1 thus can be used for both injecting the laser beams to the cell and allowing the laser beam out of the cell after 46 passes. The photographs of the beam spot pattern on the two mirrors with the red light alignment laser are shown in Figure 4d,e. By overlapping the QCL beam with the red light alignment laser, the QCL beam can also multiply reflected between these two mirrors with the same path length as the alignment laser. Once the multipass alignment procedure was accomplished, the T-shaped glass tube was installed back into the system, and it would not cause misalignment of the laser beams. After passing through the cell, the QCL beam was sent to a HgCdTe (MCT) detector and recorded by a data acquisition board (DAQ) for direct absorption spectral measurements. The total absorption length of 438.4 cm was also calibrated using the pure CH_4_ gas and the well-characterized CH_4_ transitions near 1056.79 cm^–1^.^30^ To perform the real-time ozone detection and to explore the performance of the proposed multipass enhanced photoacoustic sensor, the ozone/air gas mixtures were prepared by a homemade ozone generator with a UV lamp, as shown in Figure 5a. A 6 W UV lamp with two irradiating wavelengths of light (185 and 254 nm) was used to produce the O atoms through the photolysis of O_2_ in the air. The generated O atoms can further react with O_2_ to produce O_3_. In the experiment, the ozone generator with a volume of 9.4 L was first filled with laboratory air at atmospheric pressure, and then both inlet and outlet valves were closed. By turning on the UV lamp, waiting for a few minutes, and then turning it off, the O_3_/air mixtures could be produced with stable mixing ratios. To quantify O_3_ in the O_3_/air mixtures using direct absorption spectroscopy, the transmission spectrum of the air [*T*_air_ (ν)] was recorded as the background spectrum and the O_3_ absorbance spectrum could be derived by –ln [*T*_mix_ (ν)/*T*_air_ (ν)], in which *T*_mix_ (ν) represents the transmission spectrum of the ozone/air gas mixture. Figure 5b shows the measured absorbance spectrum of the O_3_ ν_3_ 23_0,23_–22_0,22_ and 28_2,27_–28_0,28_ transitions by employing DFB–QCL with the T-type multipass cell. Based on the known line strengths of these transitions and the total absorption path (438.4 cm), the mixing ratios of O_3_ in the ozone generator could be accurately determined. In the experiments, the ozone/air gas mixture was injected into the T-type photoacoustic cell by using a mass flow controller (MFC). For each preparation, the gas mixture in the generator could be continuously used with the stable O_3_ mixing ratios and the O_3_ concentration variations were observed less than 1% during continuous measurement at a cell pressure of 60 Torr and a flow rate of 50 sccm over 30 min. Figure 5c presents the obtained mixing ratios of generated O_3_ as a function of the adopted photolysis time in different preparation processes. The O_3_ concentrations in the T-type multipass cell could be diluted by adding another stream of air which was controlled by another MFC to perform the ozone detection with a wide range from one hundred ppm to sub-ppm levels.

**Figure 6:**
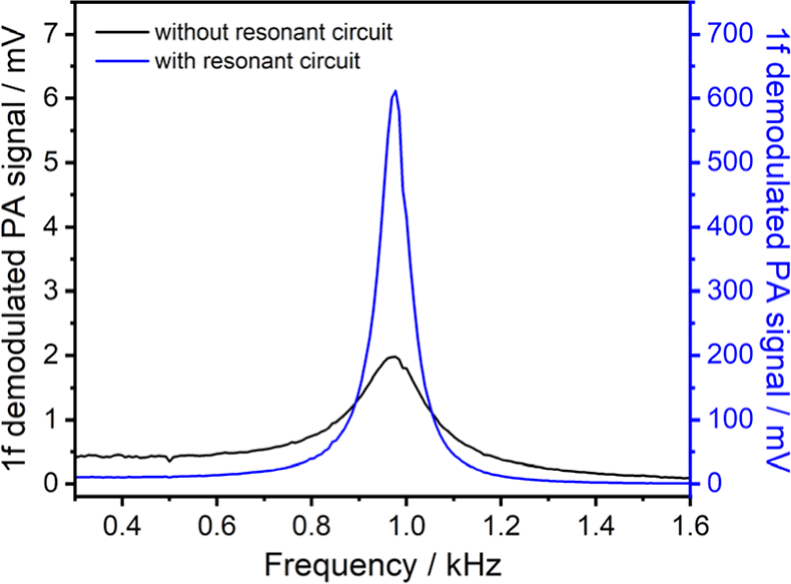
Frequency response curves of the T-type PA sensor without (black) and with (blue) the addition of the resonant circuit. Here, the flow rate of the ozone/air gas mixture is 50 sccm, the O_3_ concentration is 125 ppm, the total pressure is 60 Torr, and the temperature is 296 K. The experimental setup for the measurements of the frequency response curves is shown in Figure S3.

**Figure 7:**
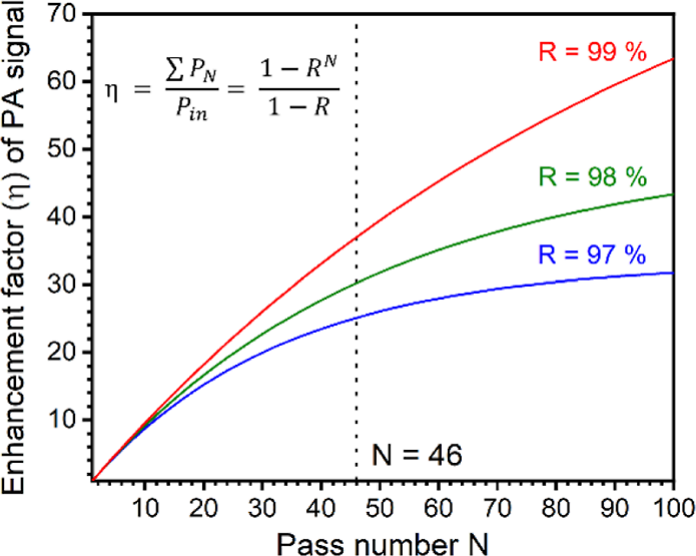
Calculated multipass enhancement factor (η) of the photoacoustic signal as a function of the pass number. Here, *P*_N_ is the optical power of the *n*th pass beam, *P*_in_ is the optical power of the incident laser (the first pass beam), *N* is the pass number, and *R* is the mirror reflectivity.

In photoacoustic (PA) spectroscopy, the intensity of the PA signal depends on the excitation light power, concentration, and absorption cross-section of the trace molecule, as well as the construction of the acoustic resonator and receiver. For the T-type PA cell, the resonant center frequency depends on the length of the resonant cylinder.^32^Figure 6 shows the measured frequency response curves of our T-type PA sensor. The first-order resonance frequency (*f*_res._) of the cell was obtained to be 973 Hz. By the addition of a resonant circuit in the acoustic receiver, the PA peak signal can be increased by a factor of 310 and the *Q*-factor of the PA signal can also be improved from 5.5 to 12.6, in which the resonant circuit was designed with a center frequency of 973 Hz and a narrow bandwidth of 11 Hz, corresponding to a *Q*-factor of 88. The experimental design and frequency response simulations of the resonant circuit are shown in Figures S1 and S2. Furthermore, the PA signal can be enhanced by increasing the pass number of the laser beam inside the absorption cavity. Figure 7 shows the multipass enhancement factor of the PA signal as a function of the pass number.

Considering the reflectivity (*R* ∼ 98%) of the used gold mirrors, the enhancement factor (η) of the PA signal was estimated to be ∼30 for the design of 46 passes of the laser beam in this work. To further perform sensitive O_3_ detection with second harmonic (2*f*) photoacoustic spectroscopy, the wavelength of the QCL was modulated by adding a sine wave to modulate the current at a modulation frequency (*f*_mod._) of 486.5 Hz, corresponding to half of the cell resonance frequency (*f*_res._). By using the lock-in amplifier, the 2*f* demodulated PA signal can be obtained and recorded by the DAQ system. Figure S4 shows the dependence of the 2*f* demodulated PA signals on the amplitudes of the modulation current. For O_3_ detection in the ozone/air gas mixtures at a total pressure of 60 Torr and 296 K, the amplitude of the modulation current of 2.3 mA was used to obtain the 2*f* demodulated PA signals with the best signal-to-noise ratio.

### Real-Time Switched Measurement of Direct Absorption and Photoacoustic Spectra

**Figure 8:**
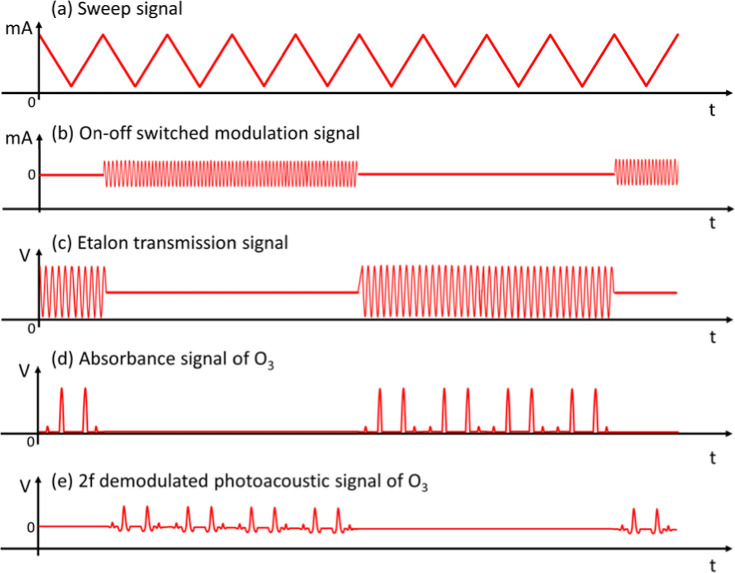
Timing schemes for (a) the laser current sweep and (b) modulation signals as well as for the data collections of (c) the etalon transmission signals, (d) the direct absorbance spectra, and (e) the 2*f* demodulated photoacoustic spectra of O_3_.

By employing the proposed multipass enhanced photoacoustic sensor, we could monitor the O_3_ signal with both the direct absorbance spectra and the 2*f* demodulated photoacoustic spectra. Figure 8 shows the schematic timing schemes for scanning and modulation of the laser current and for data acquisitions of the etalon transmission, direct absorbance, and 2*f* demodulated photoacoustic signals. To implement real-time self-calibration of the 2*f* photoacoustic signals with the direct absorbance spectra of O_3_, the laser current was continuously back-and-forth scanned with the triangle wave at a frequency (*f*_sweep_) of 1 Hz to measure the spectra in the region of 1056.87–1056.98 cm^–1^. Additionally, the current modulation signal was controlled with a periodic on/off switching signal at a frequency (*f*_switch_) of 125 mHz to perform real-time switched measurements of the direct absorption and the 2*f* demodulated photoacoustic spectra. In the experiment, the etalon transmission and the direct absorbance signals were measured when the current modulation signal was switched off, and the 2*f* demodulated photoacoustic spectra could be obtained when the laser was operated with current modulation. All these time-dependent signals were recorded by the data acquisition board (DAQ) with a sampling rate of 1 kS/s and real-time-analyzed with the LabVIEW program.

## Results and Discussion

### Self-Calibrating Ozone Detection

**Figure 9:**
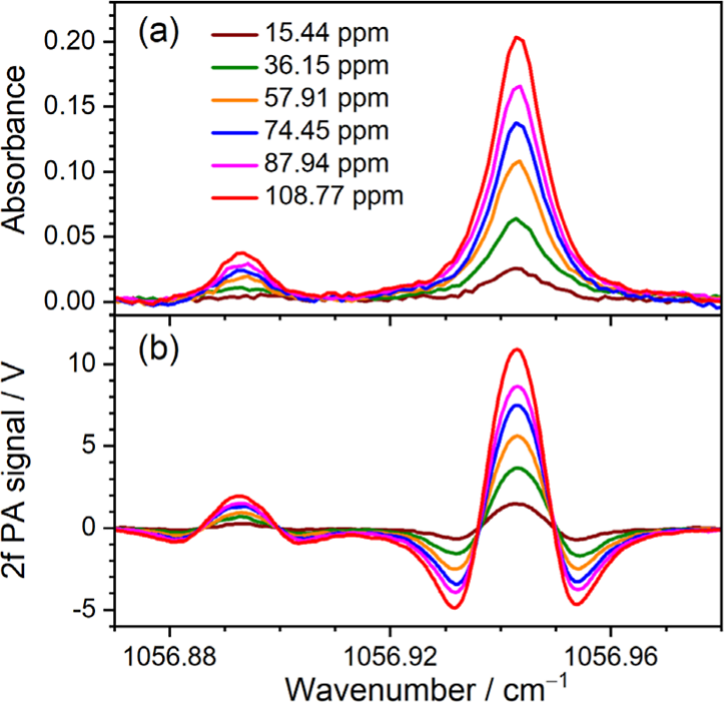
(a) The direct absorbance spectra and (b) the 2f demodulated PA spectra of O_3_ measured by the T-type multipass photoacoustic sensor with the ozone/air flow mixtures at a flow rate of 50 sccm and different O_3_ concentrations. The total pressure of the cell is 60 Torr, and the temperature is 296 K. Here, each spectrum was obtained with 16 averages.

Figure 9 shows the representative direct absorbance spectra and 2f demodulated PA spectra of the flowing gas mixtures with different O_3_ concentrations. With our experimental scheme, rapidly switching measurements between the direct absorption and PA spectra can be achieved for real-time self-calibration of the 2f PA signals with the direct absorbance spectra of O_3_.

**Figure 10:**
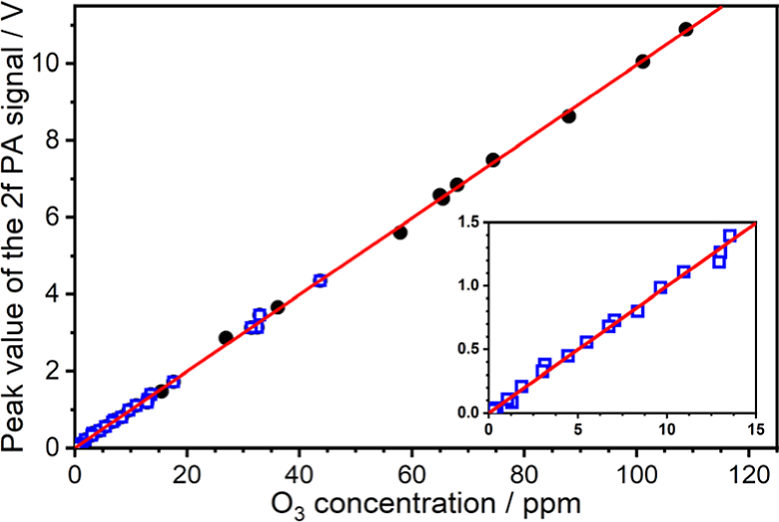
Linear relationship between the peak value of the 2*f* demodulated PA spectra at 1056.944 cm^–1^ and the O_3_ concentration. The O_3_ concentrations of black circle points were obtained by analyzing the direct absorbance spectra at the conditions of 60 Torr and 296 K. The O_3_ concentrations of blue square data were estimated by the flow rates of air and O_3_/air mixture and the determined mixing ratio of O_3_ in the ozone generator. The inset shows the zoom-in of the low O_3_ concentration region. The red line represents the linear fitting curve of the data with a slope of 99.7 mV/ppm.

Figure 10 displays the linear dependence of the 2*f* PA peak value at 1056.944 cm^–1^ on the O_3_ concentration. A linear fit of all data points was performed to obtain the PA sensor calibration curve for ozone. The fitted slope was obtained to be 99.7 mV/ppm. Considering the errors of absorption path (<1%), spectral analysis (<1%), line strength of O_3_ (2%), stability of O_3_/air gas flow (<1%), and the fitted slope of the calibration curve (0.03%), the overall uncertainty of the O_3_ concentration detection was estimated to be less than 3%, indicating that accurate O_3_ sensing with the proposed MPPA sensor system could be accomplished.

### Photoacoustic Sensor Performance Assessment

**Figure 11:**
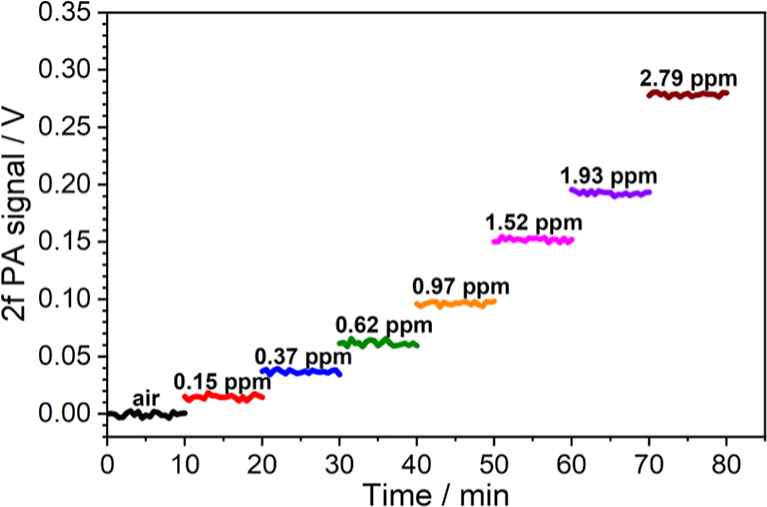
2f demodulated PA signals of O_3_ for different O_3_ concentration levels ranging from 0 to 2.79 ppm recorded as a function of time. The average time of each recording point is 30 s. Here, the total flow rate is 150 sccm, the total pressure of the cell is 60 Torr, and the temperature is 296 K.

**Figure 12:**
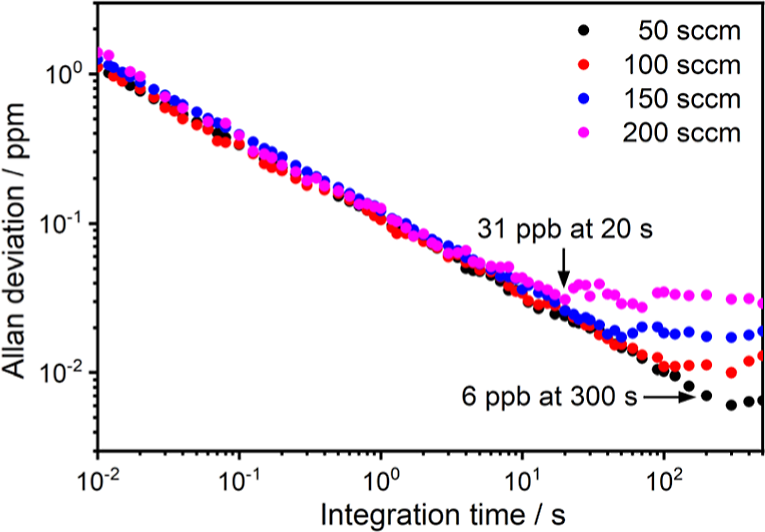
Plots of Allan deviation as a function of integration time. The noise levels were evaluated while the air was continuously flowing into the T-type multipass PA sensor with flow rates of 50, 100, 150, and 200 sccm.

**Figure 13:**
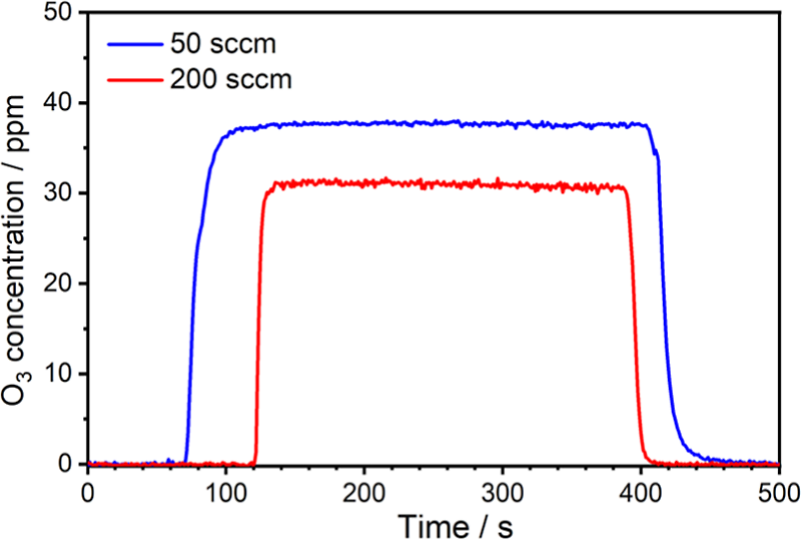
Response curves of the T-type multipass PA sensor with the gas flow rates of 50 and 200 sccm. The average time of each recording point is 1 s.

To better assess the PA sensor response and long-term sensitivity, the wavenumber of the QCL was fixed at 1056.944 cm^–1^ and the current modulation was turned on to continuously record the peak values of the 2*f* demodulated PA spectra of O_3_. Figure 11 shows the O_3_ detection for different concentration levels ranging from 0 to 2.79 ppm. For the ozone/air flow mixture with a flow rate of 150 sccm and an O_3_ concentration of 0.15 ppm, the 2*f* PA signal and the noise floor (1σ) were obtained to be 14.7 and 1.8 mV, respectively, resulting in a signal-to-noise ratio of 8.2. Accordingly, the detection limit can be estimated to be 18 ppb at an average time of 30 s. To further evaluate the noise levels of the PA sensor, a long-term signal was recorded while the air was continuously flowing into the T-type PA cell with different flow rates. Figure 12 shows the Allan variances as a function of the measurement times. For the experiment with a flow rate of 200 sccm, the detection limit of O_3_ was obtained to be 31 ppb at an average time of 20 s. By employing the flow rate of 50 sccm, the minimum detection limit of O_3_ could be achieved down to 6 ppb at an average time of 300 s, corresponding to a normalized noise equivalent absorption (NNEA) coefficient of 8.58 × 10^–9^ W cm^–1^ Hz^–1/2^ which is comparable to that of other MPPA sensor systems, as shown in Table 1. Moreover, the dynamic responses of the sensor were studied under gas flow rates of 50 and 200 sccm, as shown in Figure 13. The sensor response curves were measured by rapidly switching the flow controllers of air and the O_3_/air gas mixtures. While the flow rates were set at 50 and 200 sccm, the response times (10–90% rise or fall times) were, respectively, obtained to be approximately 16 and 7 s, validating the sensor performance for dynamic and real-time O_3_ sensing. The sensor noise level and response time can be further reduced by combining the T-type multipass PA sensor with a differential technique^26^ in the future work.

**Table 1:** Performance Comparison of Different MPPA Sensor Systems

refs	PA sensor	wavelength (μm)	molecule	power (mW)	multipass number	integration (t/s)	sensitivity	NNEA (W cm^–1^ Hz^–1/2^)
this paper	T-type cell	9.46	O_3_	30.6	46	300^b^	6 ppb	8.58 × 10^–9^
(22)	H-type cell	10.53	C_2_H_4_	6000	36	8^c^	70 ppt	2.26 × 10^–8^^d^
(25)	H-type cell	1.654	CH_4_	38.1	24	1^c^	430 ppb	9.75 × 10^–9^^d^
(26)	H-type cell^a^	1.575	H_2_S	200	30	100^b^	35 ppb	1.10 × 10^–9^
(27)	spherical cell	1.392	H_2_O	9	12	0.003^c^	80.9 ppm	2.85 × 10^–9^^d^

a Combined with differential photoacoustic spectroscopy.

b Averaging time.

c Lock-in time constant.

d Taking into account the lock-in filter slope of 6 dB/octave.

## Conclusions

In conclusion, we developed a multipass enhanced photoacoustic (MPPA) O_3_ sensor based on a T-type PA cell coupled with a mid-infrared QCL that for the first time enables O_3_ monitoring with both direct absorption and PA detection abilities. By cyclically switching the measurements of multipass absorption and PA spectra, the 2f PA signals of O_3_ can be real-time calibrated on site with the direct absorbance spectra of the O_3_/air mixtures. With an integration time of 300 s, the detection limit of O_3_ was achieved to be 6 ppb, corresponding to a NNEA of 8.58 × 10^–9^ W cm^–1^ Hz^–1/2^, in the O_3_/air mixtures with a flow rate of 50 sccm and a cell pressure of 60 Torr. Furthermore, the dynamic response of the sensor was demonstrated to validate the sensor performance in real-time O_3_ sensing which would be crucial for ozone monitoring not only in the atmosphere but also for semiconductor manufacturing^33^ and medical applications.^34^ Different trace molecules could be also probed with the proposed MPPA sensor system by changing the wavelengths of the QCL. Moreover, the proposed PA sensor with a unique capability of on-site self-calibration exhibits great potential in in situ monitoring of ozone and other important reactive molecules such as hydrogen peroxide (H_2_O_2_)^35^ and nitrous acid (HONO)^36^ without additional calibration systems and complex preparation of the standard gas sample.

## Supporting Information Available

Schematic diagram of the acoustic receiver, simulated frequency response curves of the resonant circuit with different parameters, experimental setup for determining the frequency response curves of the T-type photoacoustic sensor, and relationship between the 2*f* demodulated PA signals and the amplitudes of the modulation current (PDF)
