# Supplementary material for: Ppb-Level Self-Calibrating Ozone Detection Using a T-Type Multipass Enhanced Photoacoustic Sensor with a 9.46 μm Quantum Cascade Laser
Source: Anal Chem. 2025 Feb 4;97(6):3302–9. doi: 10.1021/acs.analchem.4c04999 (PMC11840802; doi:10.1021/acs.analchem.4c04999)
Supplement: Supplementary file 1 — ac4c04999_si_001.pdf [file ac4c04999_si_001.pdf]

Supporting Information

Ppb-Level Self-Calibrating Ozone Detection Using a T-Type Multipass Enhanced Photoacoustic Sensor with a 9.46 μm Quantum Cascade Laser

Yu-Xuan Wu[1,2] and Pei-Ling Luo[1]*

[1]Institute of Atomic and Molecular Sciences, Academia Sinica, Taipei 10617, Taiwan.
[2]Department of Chemistry, National Taiwan Normal University, Taipei, 11677, Taiwan

*corresponding author e-mail: plluo@gate.sinica.edu.tw

Table of content

Figure S1. Schematic diagram of the acoustic receiver.
Figure S2. Simulated frequency response curves of the resonant circuit with different parameters.
Figure S3. Experimental setup for determining the frequency response curves of the T-type photoacoustic sensor.
Figure S4. Relationship between the 2f demodulated PA signals and the amplitudes of the modulation current.

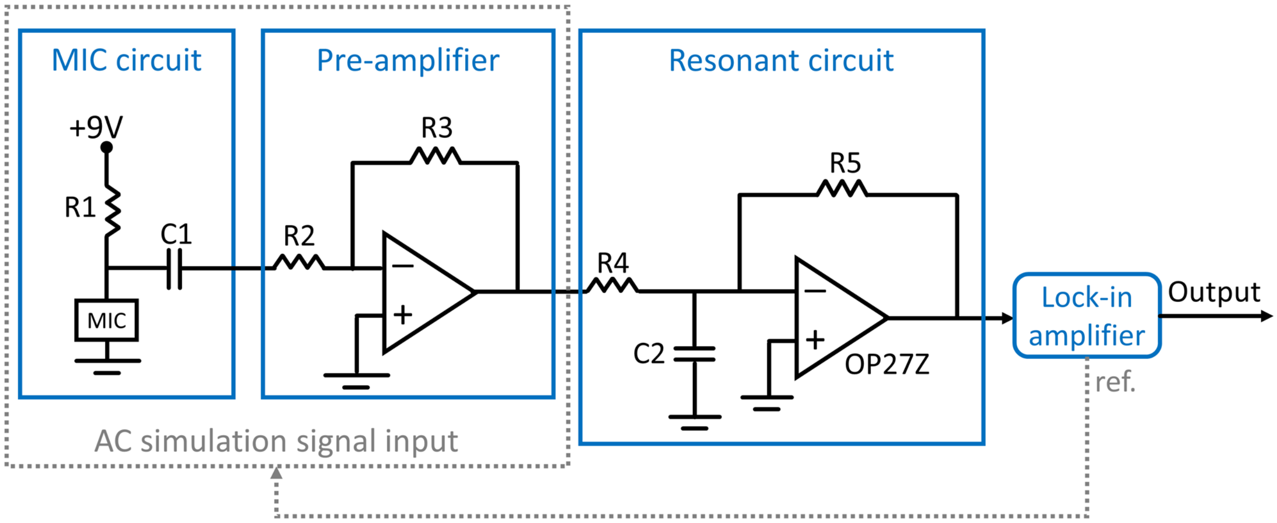

Figure S1. Schematic diagram of the acoustic receiver. The home-made resonant circuit was designed based on the low pass resonant filter circuit. Herein, the MIC is the microphone (102-4538-ND), R1 = 2.2 kΩ, C1= 1 μF, R2 =270 Ω, R3 = 540 Ω, R4 = 100 kΩ, R5 = 980 kΩ, and C2 = 1 μF. To simulate the frequency response curves of the resonant circuit, the reference signal from the lock-in amplifier was used as the AC simulation signal input to replace the part of MIC circuit and pre-amplifier.

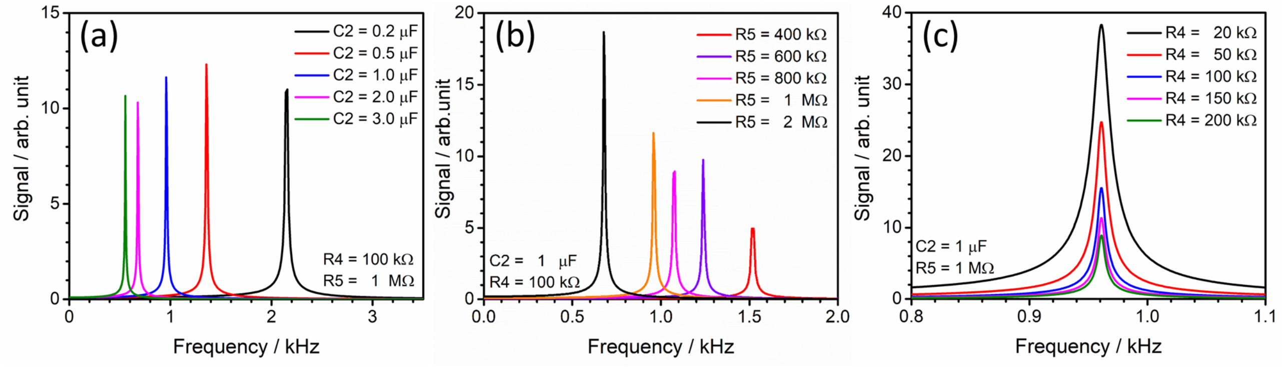

Figure S2. Simulated frequency response curves of the resonant circuit with different parameters. With the experimentally used parameters, R4 = 100 kΩ, R5 = 980 kΩ, and C2 = 1 μF, the center frequency and the bandwidth (FWHM) of the response curve were obtained to be 973 Hz and 11 Hz, respectively, resulting in the Q-factor of 88 for the resonant circuit. The Q-factor is defined as the ratio between the resonant central frequency and the full width at half-maximum (FWHM) of the frequency response curve.

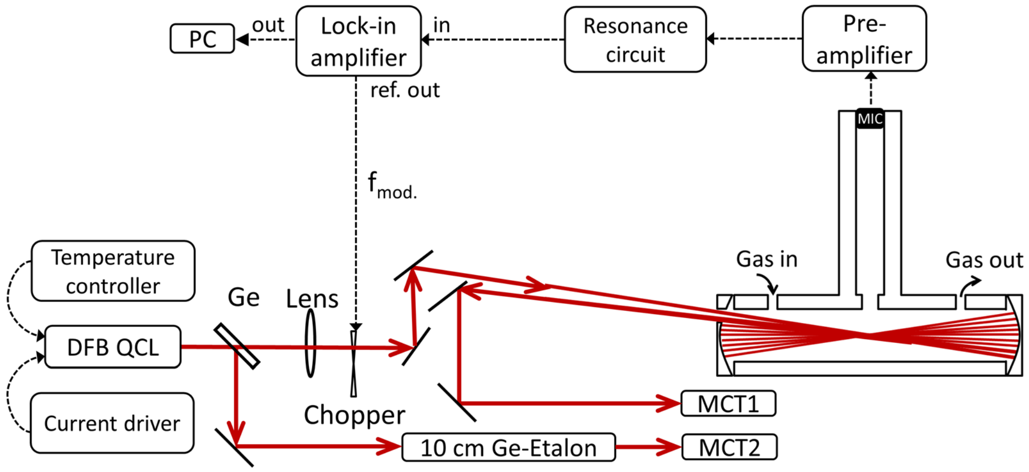

Figure S3. Experimental setup for determining the frequency response curves of the T-type photoacoustic sensor. Here, DFB-QCL is the distributed feedback quantum cascade laser and PC is the computer. The frequency response curves were obtained by step scanning the reference frequency of the lock-in amplifier.

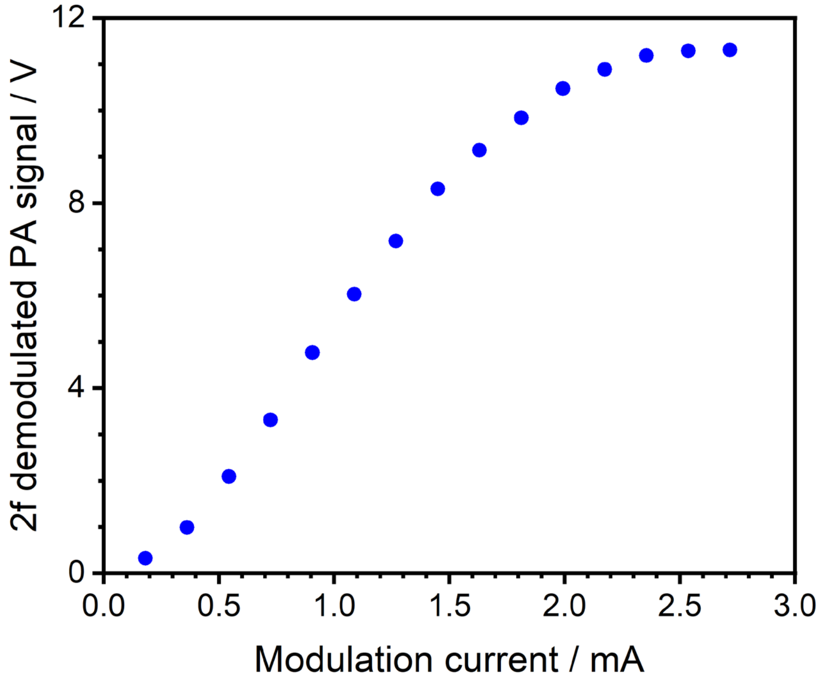

Figure S4. Relationship between the 2f demodulated PA signals and the amplitudes of modulation current. Here, the flow rate of the ozone/air gas mixture is 50 sccm, the total pressure is 60 Torr, and the temperature is 296 K.
